# The Effects of a CBT-I Based App-Program on Sleep Quality, Insomnia Severity, Psychological Strain and Quality of Life: A Pilot Study

**DOI:** 10.2147/NSS.S501633

**Published:** 2025-09-29

**Authors:** Alexandra Hinterberger, Esther-Sevil Eigl, Pavlos I Topalidis, Manuel Schabus

**Affiliations:** 1 Laboratory for Sleep, Cognition & Consciousness Research, Department of Psychology, Paris Lodron University of Salzburg, Salzburg, Austria; 2 Centre for Cognitive Neuroscience Salzburg (CCNS), Paris Lodron University of Salzburg, Salzburg, Austria

**Keywords:** insomnia, CBT-I, prevention, feasibility, digital intervention, app-program

## Abstract

**Purpose:**

Given rising numbers in sleep disorders like insomnia and insufficient availability of treatment options, the need for well-validated digital interventions rises. This pilot study aims at assessing the feasibility of an app-program which combines sleep-training based on core elements of Cognitive Behavioural Therapy for Insomnia (CBT-I) with reliable sleep-monitoring based on heart rate variability via an ECG-sensor.

**Patients and Methods:**

About 48 participants (26 females) aged 30–73 (M = 50.33 ± 11.88) were included in the study. At the beginning of the baseline (T0), at start (T1) and end (T2) of the 6-week training phase as well as 4 weeks after the end of the program (T3; follow-up) several questionnaires assessing sleep quality, insomnia severity, general psychological symptom severity, depression, anxiety as well as quality of life were completed. Furthermore, ambulatory polysomnography (PSG) was conducted three times at T0, T1 and T2. General feasibility was assessed by conducting interviews.

**Results:**

Overall, the app-program as well as the study protocol was deemed as feasible according to the participants, besides some difficulties regarding app-instructions and certain technical issues, as well as some expected complaints about worse sleep quality during PSG-recordings. For statistical results, insomnia severity (p < 0.001, r = 0.67), sleep quality (p < 0.001, r = 0.56), general psychological symptom severity (p < 0.001, r = 0.68), depression (p = 0.002, r = 0.50) and anxiety (p < 0.001, r = 0.60) improved significantly during the training phase, while quality of life [physical (p = 0.014, r = 0.41) and psychological health (p = 0.049, r = 0.35)] improved significantly during the follow-up-period. PSG data revealed a significant decrease in Wake After Sleep Onset over the course of the study (p = 0.025, r = 0.36), yet no significant changes were found for other sleep parameters.

**Conclusion:**

The app-program was largely feasible and potentially effective in improving sleep and well-being. PSG-derived WASO changes highlight the value of objective sleep measures. Future studies should refine protocols and include control conditions for greater generalizability.

## Introduction

Restorative and sufficient sleep is needed for vital processes such as physical recovery, growth of bones and muscles,^1^ the formation and strengthening of the immune system,^2^ and it is furthermore beneficial for cognitive abilities such as memory consolidation and processing of emotional stimuli.^3^ Particularly in the western world, however, more and more people are not achieving the recommended amount of sleep, as also shown in an Austrian survey: Only about half of nearly 1000 participants reported sleeping between 7 h and 9 h per night,^4^ which is the recommended amount of sleep for adults aged 18–64 years according to the National Sleep Foundation.^5^ While in 2010 sleep disturbances were reported by 18% of the Austrian population,^6^ ten years later the number was at 46%,^4^ indicating a 2.5-fold increase. This sharp and rapid augmentation illustrates the steadily growing prevalence of sleep problems such as insomnia^7^,^8^ in modern societies and asks for further attention. Affecting currently 6–12% of the population, insomnia is considered the most common sleep disorder in western societies.^9^,^10^ However, up to 30% of the general population suffer from disordered sleep and symptoms of insomnia, without necessarily meeting criteria for an insomnia diagnosis.^11^,^12^ Furthermore, only an alarming low number of 31% of the Austrian population qualify as good sleepers, according to Pittsburgh Sleep Quality Index (PSQI)^13^ self-reporting.^4^ Insufficient sleep and chronic insomnia are associated with multiple health constraints, including an increased risk for developing or dying from cardio-vascular diseases^14^ as well as for affective disorders,^15^,^16^ type 2 diabetes^17^ and substance abuse such as increased alcohol consumption.^18^ Moreover, the socio-economical costs of a person with symptoms of insomnia or an insomnia disorder are increased 3–12 times due to decreased working performance and absenteeism,^19^ resulting in annual costs of insomnia of more than $ 60 billion in the US^20^ and € 40–50 billion in Germany.^21^

The first-line treatment as suggested by the European Sleep Research Society as well as by the World Sleep Society^22^ is the Cognitive Behavioural Therapy for Insomnia (CBT-I), which includes elements such as stimulus control, sleep restriction, psychoeducation, relaxation methods and cognitive training to restructure dysfunctional thoughts.^23^ CBT-I, traditionally held in face-to-face settings, has been shown efficacious regarding improvements in the Insomnia Severity Index (ISI)^24^ and the PSQI, as well as for subjectively (and sometimes objectively) derived sleep parameters such as total sleep time (TST), sleep onset latency (SOL), wake after sleep onset (WASO) and sleep efficiency (SE) from pre- to post-treatment.^25–27^

Since the need for CBT-I offered in person cannot be met sufficiently due to a lack of trained clinicians, digital CBT-I (dCBT-I), which can be conducted without the supervision of a clinician, represents a good, additional treatment option.^28^ Additionally, not every patient is willing to consult clinical support for their sleep complaints, and dCBT-I might be a more accepted low-threshold alternative in such cases. For dCBT-I, the content of CBT-I is digitized and made available in the form of a website or smartphone application. Various studies and meta-analyses indicate good efficacy of already existing dCBT-I programs, provided by websites or smartphone-apps, in terms of improvements in ISI and sleep parameters such as TST, SOL, WASO, SE and number of awakenings (NOA) from pre-intervention to post-intervention,^29–32^ showing non-inferior effects as compared to group-delivered CBT-I,^29^ while these effects, except for the improvement of TST remained stable in follow-ups of 4 weeks to 6 months.^31^ It should be emphasized, however, that the improvement in sleep parameters refers to sleep parameters collected subjectively with a sleep diary. Objective measures of sleep using eg, polysomnography (PSG) or actigraphy are usually not performed in these studies, and so far, have not been included in the digital program itself as a form of continuous sleep monitoring. Furthermore, long-term adherence and engagement with digital CBT-I seem to pose a challenge.^33^,^34^ However, digital programs have the potential to facilitate access to insomnia treatment in the context of a stepped-care model.^35^ Additionally, incorporating artificial intelligence (AI)-driven personalization and automated feedback in digital psychotherapy programs for depression, anxiety and stress have shown promising results in enhancing user engagement and treatment outcomes.^36^

To summarize, digital CBT-I programs are a well-performing and effective complement to face-to-face CBT-I therapies, especially with regard to a stepped-care model for insomnia, while posing challenges regarding long-term engagement, which may be due to rather non-engaging approaches to dCBT-I so far. What existing and established dCBT-I programs have in common is the content which consists of sleep hygiene exercises, cognitive training, as well as techniques for behavioural changes, as well as self-monitoring in terms of a subjective sleep diary. One limitation is that the evaluation of such programs most solely relies on subjective self-reports of improved sleep quality over time. What these programs usually lack is a way to objectively and reliably record and evaluate sleep measures over the full intervention period. Objective assessment is usually disregarded since insomnia is understood to be a disorder that manifests mainly in the subjective experience, and it is argued that subjective sleep quality cannot be adequately predicted by objective measurement methods such as polysomnography.^37^ However, in addition to subjective sleep perception and its change over the course of treatment, consideration of potential changes in objective sleep parameters is worthwhile and necessary as some meta-studies do find objective sleep alterations^27^,^38^ that ask for intervention and physiological normalization. Several studies also describe a significant discrepancy between subjectively and objectively assessed sleep parameters in insomnia patients, such as an overestimation of sleep onset latency^39^ or an underestimation of total sleep time,^40^ which call into question how accurate sleep self-reports can be especially in disordered sleep patients. This discrepancy, which is typical for insomnia sufferers, can be reduced through CBT-I,^41^ and can only be made visible by collecting and comparing both subjective and objective measures. In addition, as described before, insufficient sleep is associated with a variety of physical health problems such as an increased risk of developing cardiovascular diseases,^14^ type 2 diabetes,^17^ or an increased susceptibility to infection.^42^ In order to assess these risks as well as to monitor changes in sleep across treatment, continuous and valid objective measurements of sleep are needed. Furthermore, receiving feedback on sleep might as well aid with engagement in the digital treatment program.

This study aimed at investigating the feasibility of a smartphone-based app-program that integrates a i) sleep training program based on CBT-I (over the course of 6 weeks) with ii) sleep evaluation done subjectively as well objectively using an affordable and easy-to-wear electrocardiography (ECG) chest belt, and daily sleep analysis based thereon. Since to our knowledge, the integration of objective sleep is a novel approach to digital CBT-I, the emphasis of this study was on a first evaluation of the user experience of the app-program and the study design to be used in future studies focusing on effectiveness. The focus of the present study therefore was not the evaluation of the efficacy of the app-program in the sense of a medical treatment, but rather are we here reporting effects found in retrospect on subjective changes of sleep quality, insomnia severity, psychological strain and quality of life as well as PSG-derived measures over the course of the 6-week training period.

## Materials and Methods

### Participants

**Figure 1:**
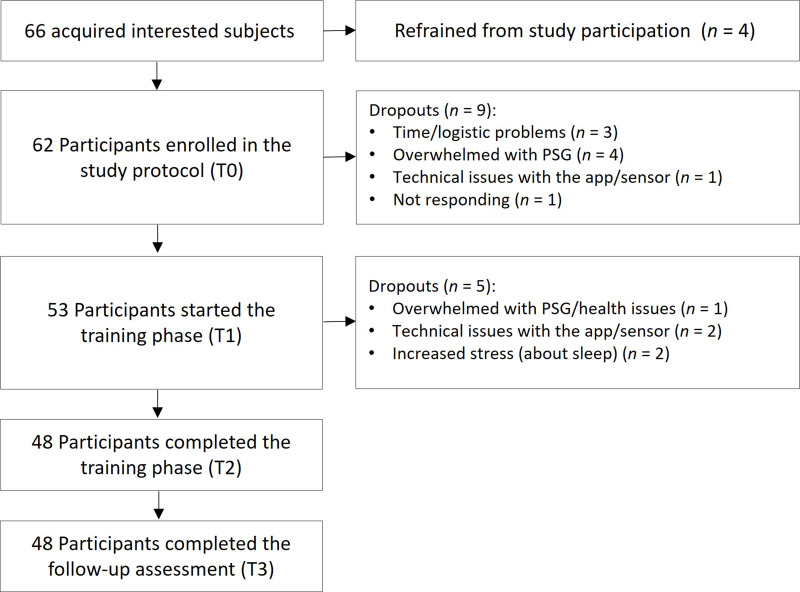
Flow of participants. Out of 66 acquired interested subjects 62 enrolled in the study protocol with 53 participants entering the 6-week training phase. A total of 48 participants completed the training phase as well as the follow-up assessment 4 weeks after the end of the training phase.

Recruitment was conducted via public media and newsletter announces. As the main purpose of this study was to evaluate the feasibility of the app program and the study protocol in general, rather than testing for hypotheses, we did not perform a priori sample size calculations but rather recruited volunteers until available resources were exhausted (ie, due to a limited number of PSG devices for parallel testing). Inclusion criteria were being at least 18 years of age, own a smartphone and live in or close to the city of Salzburg, Austria, as multiple visits at the sleep laboratory at the University of Salzburg were necessary during the study. For this first pilot study, we did not define the presence of sleep problems as a necessary inclusion criterion, yet cognizant of the fact that the majority of interested subjects would be individuals affected by disturbed sleep. The study had been approved by the ethical committee of the University of Salzburg (GZ 46/2020) and was conducted in accordance with the Declaration of Helsinki. All participants had given informed consent prior to enrolment in the study protocol. For a detailed flow of participants, please refer to Figure 1.

### Study Design and Procedure

**Figure 2:**
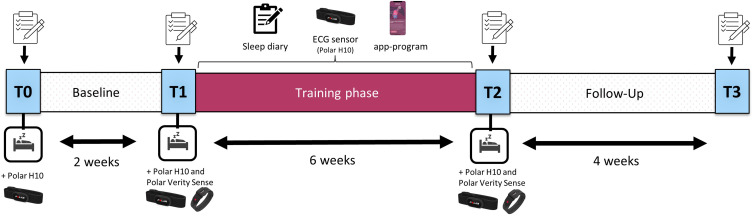
Study design. Effects of the “Virtual Sleep Lab” app-program on sleep were assessed subjectively via questionnaires at baseline, pre- and post-training and follow-up (T0-T3) as well as objectively via ambulatory polysomnography at baseline, pre- and post-training (T0-T2). Furthermore, a sleep diary was completed daily (T0-T2), and an ECG chest belt (Polar^®^ H10) was used for continuous objective sleep monitoring from pre-training (T1) to post-training (T2). The survey symbol illustrates additional questionnaire evaluations during the study (PSQI, ISI, Brief Symptom Inventory (BSI) and the WHO Quality of Life Questionnaire (WHOQOL-BREF).

In this pilot study, taking place from February to June 2022, we assessed the feasibility of the smartphone-based VSL-program (Nukkuaa^TM^, now: sleep^2^, Nukkuaa GmbH, Salzburg, Austria) during a training phase of 6 weeks, following upon a 2-week waitlist baseline (cf. Figure 2). At the beginning of the baseline (T0), at the start (T1) and end (T2) of the 6-week training phase, as well as at the follow-up (T3) 4 weeks after the end of the training, several online questionnaires were completed. The questionnaires consisted of the PSQI, the ISI, the Brief Symptom Inventory (BSI)^43^ and the WHO Quality of Life Questionnaire (WHOQOL-BREF).^44^ Additionally, three nights with ambulatory polysomnography (PSG) were conducted (T0-T2) to investigate and validate changes in objectively assessed sleep parameters. During the training phase, participants worked on the content of the VSL-program and used an ECG sensor (Polar^®^ H10, Polar Electro; Kempele, Finnland) daily to continuously monitor their sleep. Furthermore, a short sleep diary was filled in every morning to assess subjective changes in sleep quality and sleep parameters like TST, SOL or nocturnal awakenings. After the training phase, participants did not need to continue the VSL-program but were free to do so until the end of the follow-up period (T3). The approximate further usage of the program was assessed during the follow-up questionnaire, resulting in the following groups: no usage (n = 17), rare usage (1–2 days a week; n = 11), frequent usage (3–4 days a week; n = 5), regular usage (5–7 days a week; n = 15).

### Sleep Training

The training program, aiming at easing sleep problems such as insomnia, is built up in consecutive levels, which contain short daily exercises based on the core elements of CBT-I, such as psychoeducation, relaxation exercises, sleep restriction, stimulus control and cognitive training. This smartphone-delivered program was developed based on a previous digital, web-based CBT-I program from our research group.^45^ The main app-program consisted of 6 levels, which could be completed during the 6-week training phase when using the app daily. Each level consists of certain exercises such as videos and short blog posts about psychoeducation and sleep hygiene, audio files for relaxation exercises, interactive chat-bots providing cognitive therapy and sleep restriction, and sleep tips for methods of stimulus control. To complete a level and advance to the next one, certain conditions must be met: i) completing each exercise at least once, ii) filling in the sleep diary at least 5 times and iii) administering audio relaxation techniques at least 7 times. The program is structured in a way that an engagement with the app for around 10 to 15 minutes per day is sufficient to progress in the program in order to make daily usage and integration into everyday life easier for the users. Importantly, and as a novel feature as compared to our previous work that only included sleep diaries as sleep assessment,^45^ the app combines sleep training with the possibility of daily subjective and objective sleep-monitoring, by use of a sleep diary as well as an ECG sensor, which is worn at night during sleep to measure heart-rate variability (HRV). The combination of sleep training and both subjective and practically daily objective sleep measurements has to our knowledge not been done before, making the present concept interesting to be evaluated. The algorithm used in the app to enable reliable 4-class sleep-staging using the HRV signal form the ECG sensor has been validated against PSG and published accordingly.^46^,^47^ Furthermore, the app provides daily and individualized feedback based on objectively measured sleep, helping users to deeper understand their sleep patterns.

### Materials and Measures

#### Feasibility Considerations

Feasibility considerations were assessed by conducting voluntary interviews with the participants in the middle of the training phase as well as at the end of the training phase, mostly over telephone or videocall, sometimes in person. The opinions and comments from participants regarding feasibility, program rating, potential reasons for dropout, etc. were therefore assessed in a more qualitative form rather than a systematic questionnaire, to allow for a more open way of answering. Furthermore, we drew upon the analysis of questionnaires on sleep and well-being as well as PSG in order to assess a potential worsening of symptoms over the course of the study, as those results could indicate a potentially too demanding or stress-inducing app-program.

#### Subjective Assessment

##### Insomnia Severity

For the assessment of the perceived severity of insomnia symptoms, the Insomnia Severity Index (ISI)^24^ was used at four different time points (T0-T3). Seven items are answered on a 5-point Likert scale. A total sum score ranging from 0 to 28 provides information about the degree of impairment through insomnia symptoms, while higher scores indicate a higher severity of insomnia symptoms. A score from 0 to 7 is considered not clinically significant, 8 to 14 as sub-threshold insomnia, 15 to 21 as moderate clinical insomnia and a score above 21 is classified as severe clinical insomnia.

##### Sleep Quality

To measure subjective sleep quality, the Pittsburgh Sleep Quality Index (PSQI)^13^ was used at four different time points (T0-T3). It consists of 18 items which result in a global score between 0 and 21, with higher scores corresponding to a worse sleep quality. An empirical cut-off score at 5 divides between good and bad sleepers, while a score above 5 is considered as “bad sleep quality”; a score above 10 is rated as “chronic sleep disorder”.

##### Psychological Strain and Quality of Life

Furthermore, we assessed psychological strain and quality of life for a measure of the participants current life situation. For assessing psychological strain including symptoms of depression and anxiety, the Brief Symptom Inventory (BSI)^43^ was used. We investigated the global score as a measure of general psychological symptom severity and the subscales “depression” and “anxiety” as a measure of depression and anxiety, respectively.

Quality of life was measured with the short version of the WHO Quality of Life Questionnaire (WHOQOL-BREF)^44^ which differentiates into four domains: physical health, psychological health, social relationships and environment. We hereby focused on physical and psychological health analyzed and report transformed scores ranging from 0 to 100, higher values indicating higher quality of life.

#### Objective Assessment

##### Ambulatory Polysomnography (PSG)

Sleep was measured objectively using ambulatory polysomnography (PSG) including electroencephalography (EEG), electrooculography (EOG), electromyography (EMG) and ECG at three time points (T0-T2). Ambulatory PSG, ie, participants coming to the sleep laboratory in the evening to have the electrodes mounted but sleeping at home, allowed for participants to sleep in their familiar environment, and thus provided a more realistic reflection of their sleep.

PSG was carried out using ambulatory EEG systems (16-channel amplifier, Varioport) by Becker Meditec^®^, Germany, with gold cup electrodes (Grass Technologies, Astro-Med GmbH^®^, Germany). Electrodes were set up according to the international 10/20 system^48^ at positions F3, F4, C3, C4, O1, O2 with the reference and ground electrode being at positions Cz and Fz, respectively. Additionally, 2 mastoid electrodes (A1, A2), 2 EOG electrodes, 2 EMG electrodes on the chin and 3 electrodes for recording the ECG signal and the pulse rate were applied.

The recorded data were cut and processed with the Brain Vision Analyser software (version 2.2, Brain Products GmbH, Gilching, Germany): channels were re-referenced with the opposite electrode A1 or A2, a downsampling to 128 Hz was performed, and a low cut-off filter at 0.1 Hz and a notch filter at 50 Hz were applied. Sleep analyses including sleep stage classification were then performed using the Sleepware G3 Somnolyzer software (Philips Healthcare, Best, Netherlands).

### Statistical Analyses

Statistical analyses were performed using SPSS 29 (SPSS Inc., Chicago, Illinois) and R.Studio, Version 2024.09.0 (RStudio PBC, Boston, Massachusetts). Analyses were mainly based on non-parametric Friedman analyses, as of the ordinal scale of questionnaire data and as the main research question of interest in this pilot study was to evaluate changes in assessed parameters over time (T0, T1, T2, T3 for questionnaire analyses; T0, T1, T2 for PSG analyses). The significance level was set to *p* < 0.05, while *p*-values <0.10 are reported as statistical trends; for a measure of practical significance, the effect sizes η^2^ (0.010 to 0.059: small effects, 0.060 to 0.139: medium effects, larger than 0.140: large effects), Kendall’s ω (0.10 to 0.29: small effects, 0.30 to 0.49: medium effects, larger than 0.5: large effects) and *r* (0.10 to 0.29: small effects, 0.30 to 0.49: medium effects, larger than 0.5: large effects) are provided.^49^ For post-hoc comparisons, Wilcoxon-rank-sum-tests were applied. Two-tailed critical *p*-values adjusted for multiple comparisons using the Bonferroni correction are reported. For PSG analysis, extreme outliers (ie, data points deviating more than ±2 SD from the mean) were excluded from the analysis when values were considered unplausible (eg, due to a measurement error). For descriptive values of the outcome variables divided by assessment point refer to Table 1 and Table 2.

**Table 1:** Mean (M), Median (Mdn) and Standard Deviation (SD) for the Outcome Measures: Pittsburgh Sleep Quality Index (PSQI), Insomnia Severity Index (ISI), Brief Symptom Inventory (BSI) and WHO Quality of Life Questionnaire (WHOQOL-BREF) at Baseline (T0), Pre-Training (T1), Post-Training (T2) and One-Month Follow-Up (T3)

Outcome Measures: Questionnaires																					
Time	ISI			PSQI			BSI Global Severity Index			BSI Subscale Depression			BSI Subscale Anxiety			WHOQOL-BREF Domain Physical Health			WHOQOL-BREF Domain Psychological Health		
	M	Mdn	SD	M	Mdn	SD	M	Mdn	SD	M	Mdn	SD	M	Mdn	SD	M	Mdn	SD	M	Mdn	SD
T0	14.21	14.00	5.10	8.98	9.50	3.46	0.51	0.39	0.40	0.58	0.33	0.59	0.52	0.50	0.46	69.48	69.00	15.17	64.56	69.00	14.34
T1	13.85	13.00	4.65	8.29	8.00	2.88	0.53	0.43	0.38	0.570	0.50	0.59	0.53	0.50	0.43	70.04	69.00	15.76	64.79	69.00	16.07
T2	10.67	10.00	4.83	6.79	6.00	2.85	0.37	0.33	0.30	0.40	0.33	0.40	0.32	0.17	0.36	71.58	75.00	16.08	66.77	69.00	15.13
T3	9.90	9.00	4.84	6.27	5.50	3.15	0.35	0.26	0.31	0.38	0.25	0.43	0.29	0.17	0.29	75.46	75.00	15.00	69.73	69.00	15.17

**Note**: N = 48.

**Table 2:** Mean (M), Median (Mdn) and Standard Deviation (SD) for the PSG-Derived Outcome Measures: Time in Bed (TIB), Total Sleep Time (TST), Sleep Onset Latency (SOL), Wake After Sleep Onset (WASO) and Sleep Efficiency (SE) at Baseline (T0), Pre-Training (T1) and Post-Training (T2)

Outcome Measures: Polysomnography															
Time	TIB (in min)			TST (in min)			SOL (in min)			WASO (in min)			SE (in %)		
	M	Mdn	SD	M	Mdn	SD	M	Mdn	SD	M	Mdn	SD	M	Mdn	SD
T0	463.00	461.50	69.19	375.52	364.30	58.26	12.51	8.50	10.81	74.96	60.00	53.47	81.64	83.85	9.43
T1	448.19	446.60	65.35	371.92	369.45	58.97	17.33	12.75	15.78	58.93	45.25	39.94	83.29	85.00	8.54
T2	450.77	464.45	62.45	372.10	371.85	62.70	25.66	10.25	44.48	53.01	47.25	34.45	82.84	84.85	10.22

**Note**: N = 38.

## Results

### Sample Characteristics

The final sample consisted of 48 participants (26 females) aged 30–73 (M = 50.33 ± 11.88), who finished the whole study protocol including the follow-up measurement. With initially 66 recruited participants (34 females; aged 30–74 years [M = 50.27 ± 12.44]), this resulted in a dropout rate of 27%, which is significantly less compared to dropout rates of other studies investigating the efficacy of digital treatment programs for insomnia in outpatient samples (34.4% dropout in Yeung et al;^50^ 40% dropout in Peter et al^51^). The majority of the sample (58.3%; n = 28) had a university degree, further 14.6% (n = 7) had graduated from high school, and 10.4% (n = 5) had gone to secondary school. The remaining 16.7% had undergone educational training (8.3%, n = 4), went to vocational school (6.3%, n = 3) or primary school (2.1%, n = 1). Although we did not explicitly define sleep problems as an inclusion criterion for this pilot study, in more than 80% of participants we found a PSQI score of above 5 at the beginning of the study, indicating bad sleep quality. For a detailed flow of participants, please refer to Figure 1.

### Subjective Assessment

#### Insomnia Severity (ISI)

**Figure 3:**
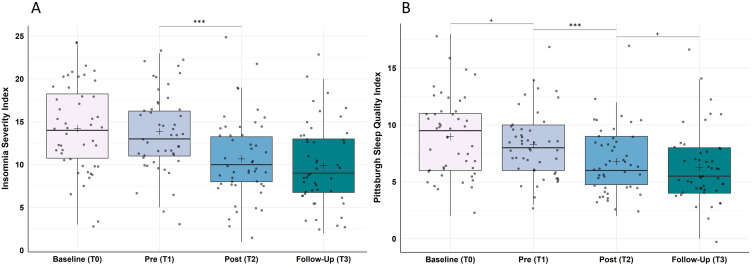
Improvement in (**A**) the severity of insomnia symptoms assessed by the Insomnia Severity Index and (**B**) the subjective sleep quality assessed by the Pittsburgh Sleep Quality Index. (**A**) Insomnia severity improved significantly from pre-training (T1) to post-training (T2) and remained stable until one-month follow-up (T3); N = 48. (**B**) Sleep quality improved tendentially from baseline (T0) to pre-training and significantly from pre-training (T1) to post-training (T2) and was by trend lower in the one-month follow-up (T3); N = 48. Higher values represent a stronger impairment in the respective measure. Horizontal lines represent the medians, boxes the interquartile range, with whiskers depicting the 1.5 interquartile range. The black cross corresponds to the mean. Asterisks indicate significance: ***p < 0.001, ^+^*p* < 0.10.

Analysis of the “ISI” showed statistically significant changes in insomnia severity over the course of the study (Χ^2^(3) = 49.44, p < 0.001, ω = 0.34). Post-hoc analyses revealed, as expected, no significant changes from baseline to pre-training (Z = −0.81, p > 0.999, r = 0.12), however, a significant improvement from pre- to post-training (Z = −4.67, p < 0.001, r = 0.67) and the results remained stable from post-training to the follow-up assessment one month later (Z = −1.55, p = 0.362, r = 0.22; cf. Figure 3A).

#### Sleep Quality (PSQI)

Analysis of the “PSQI” showed statistically significant changes in sleep quality over the course of the study (Χ^2^(3) = 46.21, p < 0.001, ω = 0.32). Post-hoc analyses revealed a statistical trend towards an improvement from baseline to pre-training (Z = −2.27, p = 0.069, r = 0.33), a significant improvement from pre- to post-training (Z = −3.85, p < 0.001, r = 0.56) and there was a trend for further improvements from post-training to the follow-up assessment one month later (Z = −2.14, p = 0.097, r = 0.31; cf. Figure 3B).

#### Psychological Strain (BSI – Global Score GSI)

**Figure 4:**
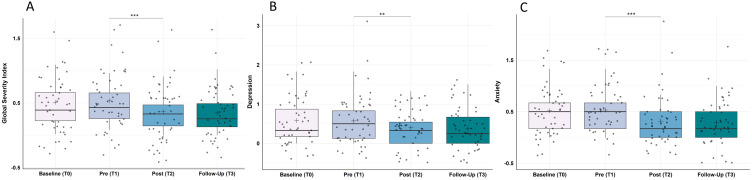
Improvement in (**A**) psychological strain measured by the Global Severity Index of the Brief Symptom Inventory (BSI), (**B**) the BSI-subscale depression and (**C**) the BSI-subscale anxiety. (**A**) Psychological strain, (**B**) symptoms of depression and (**C**) symptoms of anxiety improved significantly from pre-training (T1) to post-training (T2) and remained stable until one-month follow-up (T3); N = 48. Higher values represent a stronger impairment in the respective measure. Horizontal lines represent the medians, boxes the interquartile range, with whiskers depicting the 1.5 interquartile range. The black cross corresponds to the mean. Asterisks indicate significance: ***p < 0.001, **p < 0.010.

Analysis of the BSI global score “GSI” showed statistically significant changes in the participant’s psychological strain over the course of the study (Χ^2^(3) = 32.12, p < 0.001, ω = 0.22). Post-hoc analyses revealed no significant change from baseline to pre-training (Z = −1.42, p = 0.466, r = 0.20), yet a significant improvement from pre- to post-training (Z = −4.69, p < 0.001, r = 0.68) and results remained stable from post-training to the follow-up assessment one month later (Z = −0.64, p > 0.999, r = 0.09; cf. Figure 4A).

#### Depression (BSI – Subscale Depression)

Analysis of the BSI subscale “depression” showed statistically significant changes over the course of the study (Χ^2^(3) = 12.01, p = 0.007, ω = 0.08). Post-hoc analyses revealed no significant change from baseline to pre-training (Z = −0.17, p > 0.999, r = 0.02). There was, however, a significant change from pre- to post-training (Z = −3.44, p = 0.002, r = 0.50) and results remained stable from post-training to the follow-up assessment one month later (Z = −0.67, p > 0.999, r = 0.10; cf. Figure 4B).

#### Anxiety (BSI – Subscale Anxiety)

Analysis of the BSI subscale “anxiety” showed statistically significant changes over the course of the study (Χ^2^(3) = 34.70, p < 0.001, ω = 0.24). Post-hoc analyses revealed, as expected, no significant changes from baseline to pre-training (Z = −0.89, p > 0.999, r = 0.13), yet significant improvements from pre- to post-training (Z = −4.13, p < 0.001, r = 0.60) and results remained stable from post-training to the follow-up assessment one month later (Z = −0.56, p = 1.00, r = 0.08; cf. Figure 4C).

#### Quality of Life (WHOQOL-Bref)

##### Domain Physical Health

**Figure 5:**
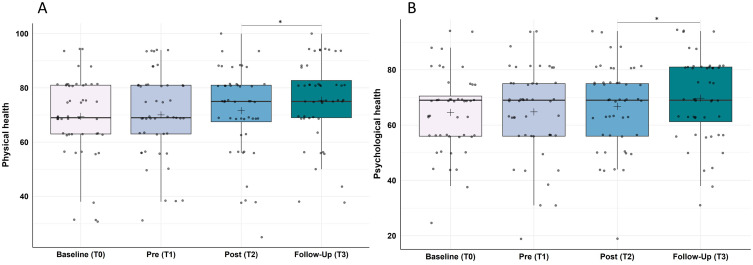
Improvement in (**A**) physical health and (**B**) psychological health measured by the WHO Quality of life questionnaire (WHOQOL-BREF). (**A**) Physical health and (**B**) psychological health improved significantly from post-training (T2) to follow-up (T3); N = 48. Lower values represent a stronger impairment in the respective measure. Horizontal lines represent the medians, boxes the interquartile range, with whiskers depicting the 1.5 interquartile range. The black cross corresponds to the mean. Asterisks indicate significance: *p < 0.050.

Analysis of the WHOQOL-Bref domain “physical health” showed statistically significant changes over the course of the study (Χ^2^(3) = 18.96, p < 0.001, ω = 0.13). Post-hoc analyses revealed no significant changes from baseline to pre-training (Z = −0.66, p > 0.999, r = 0.10), nor from pre- to post-training (Z = −0.21, p > 0.999, r = 0.03), but a significant improvement from post-training to the follow-up assessment one month later (Z = −2.83, p = 0.014, r = 0.41; cf. Figure 5A).

##### Domain Psychological Health

Analysis of the WHOQOL-Bref domain “psychological health” showed statistically significant changes over the course of the study (Χ^2^(3) = 14.96, p = 0.002, ω = 0.10). Post-hoc analyses revealed no significant changes from baseline to pre-training (Z = −0.37, p > 0.999, r = 0.05), nor from pre- to post-training (Z = −0.91, p > 0.999, r = 0.13), but a significant improvement from post-training to the follow-up assessment one month later (Z = −2.41, p = 0.049, r = 0.35; cf. Figure 5B).

### Objective Assessment

#### Ambulatory Polysomnography (PSG)

**Figure 6:**
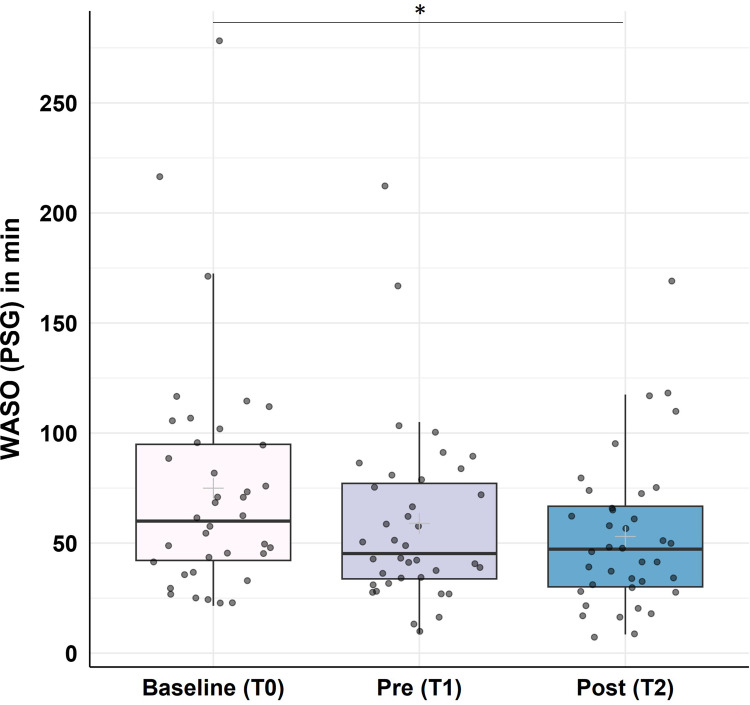
Improvement in PSG-derived Wake After Sleep Onset (WASO) in minutes. WASO decreased significantly from baseline (T0) to post-training (T2), N = 38. Horizontal lines represent the medians, boxes the interquartile range, with whiskers depicting the 1.5 interquartile range. The grey cross corresponds to the mean. Asterisks indicate significance: *p < 0.050.

Analysis of main PSG-derived sleep parameters (Time in Bed (TIB), TST, SOL, WASO, SE), data revealed significant changes in WASO (Χ^2^(2) = 6.04, p = 0.049, ω = 0.08) Post-hoc analyses revealed no significant change from baseline to pre-training (Z = −0.19, p = 0.233, r = 0.03) or pre-training to post-training (Z = −0.97, p = 0.334, r = 0.16), however, a significant decrease from baseline to post-training (Z = −2.24, p = 0.025, r = 0.36; cf. Figure 6).

Regarding TIB (Χ^2^(2) = 1.23, p = 0.540, ω = 0.02), TST (Χ^2^(2) = 1.00, p = 0.607, ω = 0.01), SOL (Χ^2^(2) = 1.97, p = 0.373, ω = 0.03), and SE (Χ^2^(2) = 3.19, p = 0.203, ω = 0.04), no significant changes were found over the course of the study.

### Usage of the Program Within the Follow-Up Period

Furthermore, we analyzed if different frequencies in the usage of the app-program within the follow-up period resulted in different outcomes in terms of changes in the various questionnaires from T0-T3, T1-T3 and T2-T3. Hereby, we used non-parametric Kruskal–Wallis-tests to investigate the differences between the 4 groups (no usage (n = 17), rare usage (1–2 days/week; n = 11), frequent usage (3–4 days/week; n = 5), regular usage (5–7 days/week; n = 15)).

No significant differences between groups were found in PSQI changes T0-T3 (H(3) = 2.29, p = 0.515, η^2^ = −0.02), T1-T3 (H(3) = 5.83, p = 0.120, η^2^ = 0.06) and T2-T3 (H(3) = 2.97, p = 0.396, η^2^ = −0.00), in ISI changes T0-T3 (H(3) = 0.73, p = 0.867, η^2^ = −0.05), T1-T3 (H(3) = 1.48, p = 0.688, η^2^ = −0.03) and T2-T3 (H(3) = 6.02, p = 0.111, η^2^ = 0.07), in BSI global score changes T0-T3 (H(3) = 1.27, p = 0.736, η^2^ = −0.04), T1-T3 (H(3) = 1.54, p = 0.673, η^2^ = −0.03), T2-T3 (H(3) = 1.18, p = 0.758, η^2^ = −0.04), in BSI subscale depression T0-T3 (H(3) = 1.72, p = 0.632, η^2^ = −0.03), T1-T3 (H(3) = 4.67, p = 0.197, η^2^ = 0.04), T2-T3 (H(3) = 3.70, p = 0.296, η^2^ = 0.02), in BSI subscale anxiety T0-T3 (H(3) = 1.05, p = 0.788, η^2^ = −0.04), T1-T3 (H(3) = 1.52, p = 0.678, η^2^ = −0.03), T2-T3 (H(3) = 1.17, p = 0.759, η^2^ = −0.04), in WHOQOL-BREF domain “physical health” T0-T3 (H(3) = 0.90, p = 0.824, η^2^ = −0.05), T1-T3 (H(3) = 0.34, p = 0.953, η^2^ = −0.06), T2-T3 (H(3) = 1.00, p = 0.803, η^2^ = −0.05), and in WHOQOL-BREF domain “psychological health” T0-T3 (H(3) = 1.23, p = 0.746, η^2^ = −0.04), T1-T3 (H(3) = 1.29, p = 0.733, η^2^ = −0.04). For changes in WHOQOL-BREF domain “psychological health” a statistical trend was observed for T2-T3 (H(3) = 7.47, p = 0.058, η^2^ = 0.10). For an overview on descriptive statistic values please refer to Supplementary Tables S1–S7.

### Factors Predicting Improvements During the Training Phase

A multiple linear regression model was conducted to assess potential predictors for the improvement of insomnia symptoms during the app training phase, defined by the difference of the ISI scores from T1 to T2. The following predictors were included in a forced entry model: Sex (male vs female), age, education (higher, ie, graduated from high school or university degree, vs lower), last completed level at T2, PSQI at T1 (low, ie, 0–5 points vs high, ie, 6 or more points), ISI at T1 (low, ie, 0–7 points, vs high, ie, 8 or more points). Analysis revealed two significant predictors: Age (b = −0.10, t (47) = −2.09, p = 0.043, 95% CI [−0.19, −0.00]) and the last complete level at T2 (b = 0.75, t (47) = 2.38, p = 0.022, 95% CI [0.11, 1.39]), indicating, that people showed higher improvement when they were of younger age and have had completed more levels of the program. The overall regression was statistically significant (R^2^ = 0.31, F (6, 41) = 3.04, p = 0.015), showing that the predictors accounted for 31% of the variability in the improvement in ISI scores (cf. Table 3).

**Table 3:** Statistical Overview Over the Coefficients Included in the Multiple Regression Model to Assess Possible Predictors Regarding Improvements in Insomnia Severity (ISI) from Pre-Training (T1) to Post-Training (T2)

Dependent Variable: ISI Change T1-T2							
Coefficients	b	SE	β	T	p	95% CI	
						LL	UL
(Constant)	2.806	3.826		0.733	0.468	−4.921	10.533
Sex	−0.571	1.127	−0.071	−0.506	0.615	−2.848	1.706
Age	−0.096	0.046	−0.282	−2.09	0.043	−0.189	−0.003
Education (binominal)	−1.601	1.223	−0.178	−1.310	0.198	−4.070	0.868
Last complete level at T2	0.751	0.315	0.330	2.381	0.022	0.114	1.388
PSQI T1 (binominal)	0.854	1.880	0.083	0.454	0.652	−2.942	−2.942
ISI T1 (binominal)	4.189	2.567	0.289	1.632	0.110	−0.996	−0.996

**Notes**: N = 48, R^2^ = 0.308, corr. R^2^ = 0.207, F (6, 41) = 3.04, p = 0.015. Education, PSQI T1 and ISI T1 were included as binominal variables: Education: 0 = education below high school graduation, 1 = education high school graduation and higher (university degree etc.); PSQI T1: 0 = sum scores of 0–5 (“good sleep quality”), 1 = sum scores of 6–21 (“poor sleep quality”); ISI T1: 0 = sum scores of 0–7 (“no clinically relevant insomnia symptoms”), 1 = sum scores of 8–28 (“at least subthreshold insomnia”).

**Abbreviations**: ISI, Insomnia Severity Index; LL, lower limit; PSQI, Pittsburgh Sleep Quality Index; T1, pre-training; T2, post-training; UL, upper limit.

## Discussion

The focus of this pilot study was to assess the feasibility of the app-program and the study protocol, as we intended to use both in future studies for the assessment of efficacy and training effects. Interviews were conducted via telephone in the middle and at the end of the 6-week training phase to acquire participants’ experiences with the digital program and the study protocol. Out of 48 people in the final sample 40 participated in both interviews, 6 in one of two interviews, and 2 refrained from participating in the interviews.

The app-based CBT-I program was well received by the majority of participants, who found the sleep tracking and feedback particularly engaging. However, some participants reported discrepancies between their perceived sleep quality and the app’s analysis, which may be due to the evolving algorithm as well as sleep discrepancy, a phenomenon frequently observed in individuals with insomnia.^41^ While many participants found the program useful, those with milder sleep disturbances were less engaged, often completing it primarily for study participation rather than personal interest.

The daily relaxation exercises were perceived as demanding, with several participants expressing a preference for greater flexibility, such as selecting from multiple audio options. Additionally, some participants did not initially realize they could change the narrator’s voice, which affected their experience. Regarding retention, two participants withdrew due to increased stress from focusing on their sleep issues, while others felt overwhelmed by the polysomnography (PSG) setup. The latter group included individuals with mild sleep disturbances and those with severe insomnia, for whom the PSG equipment further disrupted sleep. Furthermore, the structure of the CBT-I program was not entirely intuitive, with several participants only realizing after a few weeks or through study staff that progressing to the next level required fulfilling specific conditions, such as listening to relaxation exercises daily. While many participants understood the importance of regular app use for research purposes, they expressed a desire for more flexibility in choosing their engagement level. These insights highlight potential areas for refinement in future iterations of the program, particularly regarding user autonomy and guidance.

An additional factor influencing participants’ experience with the program and study retention was their initial motivation for participation. Individuals who enrolled seeking a new treatment option for long-standing sleep problems exhibited higher levels of engagement and persistence in completing the program, whereas those who participated out of general interest in a sleep study demonstrated lower levels of commitment. Given that sleep disturbances were not a strict prerequisite for inclusion, the varying degrees of personal investment likely impacted adherence and perceived usefulness of the intervention. Future studies may benefit from considering participants’ baseline motivation and tailoring engagement strategies accordingly.

This pilot study also assessed changes in subjective sleep quality, insomnia severity, psychological strain and quality of life, as well as objective, PSG-derived sleep parameters. Analysis of changes in the sleep-related questionnaires PSQI and ISI showed statistically significant improvements in subjective sleep quality as well as in insomnia severity, including symptoms such as difficulties falling asleep, maintaining sleep and early awakenings. These improvements occurred between the expected measurement time points, that is from pre- to the post-training 6 weeks later, and sometimes with even further improvements to the follow-up (4 weeks later). This indicates that subjective improvements in both clinical questionnaires can be observed after a 6-week sleep training using the VSL-program.

Specifically, ISI-scores improved from a mean score of 14.21 (Mdn = 14.00) at baseline to a mean score of 9.90 (Mdn = 9.00) at follow-up. Both scores are assigned to the category “low-threshold insomnia” according to the manual, which is defined by a score range of 8–14.^24^ Thus, despite a statistical improvement, there was on average no clinical improvement to the aspired category of “non-significant insomnia”, which is categorized by a score range of 0 to 7 points in the ISI.^24^ However, while at the start of the baseline (ie, T0) only 6.3% of the participants fell into the category of a “non-significant insomnia”, by the end of the training phase (ie, T2) 20.8% did, as well as 35.4% by the one-month follow-up (ie, T3). The number thus tripled by the end of the training phase and was even 6-times higher by the follow-up-assessment.

Similarly, PSQI-scores improved from a mean score of 8.98 (Mdn = 9.50) at baseline to a mean score of 6.27 (Mdn = 5.50) at follow-up. Both scores fall into the category of “poor sleep quality”, which is defined by a score range of 6 to 10. Values of 5 or less would correspond to good sleep quality.^52^ Thus, again, there was on average no clinical improvement in terms of changes in the sleep quality category of the PSQI. While at the beginning of the baseline (ie, T0) only 16.7% had been defined as a “good sleeper” according to the PSQI global score, after the training phase (ie, T2) 35.4% of the participants were scoring ≤5 on the PSQI global score, as well as 50% at the one-month follow-up (ie, T3) and can therefore be defined as “good sleepers” after administration of the VSL-program. The number of “good sleepers” thus doubled by the end of the program and even tripled by the follow-up-assessment. It is relevant to mention that in this pilot study, no clinical sample was recruited, but we simply recruited from the general population using public announcements in Austrian TV and radio. This fact is also reflected in the mean values of the sample in the ISI described in the last paragraph, which were, on average, also only in the range of “subthreshold insomnia” at the beginning of the study (Mdn = 14.00). It is believed that stronger improvements would have been shown if the sample on average had had higher insomnia symptoms by beginning of the study.

Another point to consider is the program progress during the training phase of 6 weeks: The objective was to complete the first 6 levels of the VSL-program, which is considered the core program of the training. In fact, only 12.5% of the sample completed these 6 levels, while another 31.3% completed the program including the 5th level. That means that only 21 individuals and thus less than half of the final sample completed at least most of the core program (including level 5). One reason for this low completion rate turned out to be the rather strict conditions that had to be met in order to advance to the next program level. Specifically, one of those conditions was to listen to the relaxation audio exercise at least 7 times, while only one time was counted per day. In our study design where one level was meant to be worked on within one week, this equaled to a requirement of daily listening to the relaxation exercises in order to complete the main program (ie, 6 levels) within the 6-week training phase. The low completion rate showed us that this requirement was set too strict, since this resulted in being the main reason of delayed advancements in the app program. A more flexible approach, eg, listening to it 5–6 times per level, or giving participants more time to complete the 6 levels would have probably been more realistic to incorporate in day-to-day life. Furthermore, we did not give an extensive introduction to the app-program in order to assess how intuitive the usage of the program is. As many participants did not realize from the beginning that certain conditions have to be met in order to advance in the program (ie, the daily completion of audio relaxations), the progress delay could not be caught up in the remaining weeks. Additionally, we also encountered some technical issues regarding the counting of the relaxation exercises. This resulted in a few cases that participants in fact listened to the audio, however, this was not saved and therefore not counted in the app. These circumstances are believed to be the main reason for progress delay and therefore the low completion rates.

In summary, it is expected that participants on average would have shown greater improvements if more individuals had completed the core VSL-program. Additional analyses support this assumption: it was shown that the further the progress in the program, the greater the improvement in terms of alleviation of insomnia symptoms during the training phase. These results suggest greater effectiveness of the training program with consistent use of the app (cf. Table 2). These findings are in line with Zachariae et al,^32^ who showed in their meta-analysis that longer treatment duration was associated with greater effect sizes.

Furthermore, there were also improvements in the other questionnaires assessed in this study. Specifically, we found improvements in general psychological symptom severity, symptoms of depression and anxiety, and an increase in quality of life in terms of perceived physical and psychological health over the course of the study.

Regarding the questionnaire data, it is interesting to note that the different frequencies of continued usage of the app-program between the end of the training phase and the follow-up did not have an influence on the improvement. Specifically, this means that people who continued to use the program did not show greater improvements at follow-up than people who did not. It was believed that individuals who continued to use the VSL-app (frequently or regularly) would benefit more than individuals who stopped using the program after the training phase. The results suggest that the main efficacy and thus impact on subjective parameters was already achieved in the first 6 weeks or during the training phase, no matter the time spent in the app-program during the follow-up period. It can further be assumed that participants who voluntarily used the app more frequently during the follow-up period were inherently more motivated and have likely used the app more consistently during the initial training phase. Consequently, these individuals may have experienced greater improvements compared to participants who engaged with the app less frequently and therefore did not achieve significant progress, regardless of the amount of later voluntary usage.

Objective, PSG-derived data revealed no significant changes in TIB, TST, SOL and SE, yet, a significant reduction in WASO from an average of 75 minutes to 53 minutes over the course of the study. This is still far from the objective WASO of healthy sleepers (around 38 minutes according to Baglioni et al),^27^ however, a reduction by 22 minutes can arguably be considered a substantial change. As objective changes are usually less pronounced and/or take more time to happen,^38^ one can assume, that potentially even greater improvements in WASO (and/or changes in other sleep parameters) could have been achieved if a follow-up PSG had been conducted. Additional PSG recordings, however, were not included in the study design due to resource- and feasibility reasons, making it impossible to test this assumption at present.

## Limitations

With regard to the limitations of the study, some essential points should be noted: In this pilot study, no control group was implemented, yet a non-training baseline and follow-up period. Therefore, it cannot be investigated in more detail whether the effects that were shown in the sample regarding an improvement in subjective sleep quality as well as an alleviation of insomnia severity are actually intervention-specific effects, or whether these also could be placebo effects. As the literature shows, and what we also observe as trend in our data subjective improvements sometimes can occur by minimal intervention^53^ or even mere inclusion in a study protocol, even though no training or therapy has yet been started.^54^ Although such improvements are good for the patient, they cannot be considered treatment-specific and accordingly need to be clearly named as such. In fact, a follow up study on the VSL program, including a waitlist control group, found that the subjective improvements in ISI and PSQI are indeed training-specific and were not found in the control group.^55^

As this was the first study of the VSL-program, sometimes technical issues were still present. For example, the type of feedback on NOA and WASO in the app was changed while the study was running. Also, the daily relaxation exercises for program advancements were not counted in certain circumstances, which for some participants resulted in delays in the progression to the next sleep training “level”. These disruptive factors may have further prevented subjects from fully engaging with the app and may have led to less trust in the digital program.

With a sample size of 48 people, we tried to allow first reliable inferences about the efficacy of the VSL-program and over the course of the 12-week study period. The present sample size may be considered too small in order to produce generalizable results regarding efficacy, especially without the inclusion of a control condition, such as a waitlist control group, however it is to be noted that the study protocol was rather resource-intensive with three PSG recordings per participant, and the inclusion of more participants would have outweighed the appropriate resources for a pilot study with a focus on feasibility and not efficacy. For replication and better generalizability of the results, it is highly needed to add further efficacy studies with larger samples, and of varying, especially more severe, degrees of sleep problems.

In general, it should be noted that due to the nature of the present study, ie, a pilot study, statements on efficacy should be handled with care, as the main focus of such studies is on feasibility and not treatment or training effects. Since pilot studies, such as the present work, tend to be of a more exploratory nature and less controlled, eg, by including a control condition or waitlist condition, results on efficacy should not be interpreted as robust effects. In the present work, those results were also used to assess a potential worsening of symptoms over the course of the study protocol, as this would have been an indicator for a potentially too demanding or stress-inducing program. Since the results did not show adverse effects on sleep and well-being, those apprehensions were not confirmed.

## Conclusion

In summary and considering the points raised, it can be stated that the VSL-program, with its concept of combining CBT-I-oriented sleep training together with continuous objective sleep monitoring, as well as the study design is to most extend feasible, and the app-program furthermore potentially effective in improving subjective sleep parameters, components of quality of life and comorbid symptoms such as depression and anxiety. Furthermore, and taking into account the substantial change in PSG-derived WASO, it can be concluded, that investigating not only subjective, yet also objective measures of sleep is worthwhile, as they provide a deeper understanding of the potential effects of digital CBT-I. However, for future studies it is important to take into consideration the insights regarding the feasibility of the app-program and the study protocol. Specifically, a less strict conditions in order to advance in the program, clearer instructions regarding the app-program, as well as allowing more time to complete the program may foster greater advancement and therefore higher improvements in sleep disturbed people. In order to produce generalizable and robust findings, future studies should furthermore include control conditions as well as include people with more severe degrees of sleep complaints. This pilot study was conducted in order to assess the feasibility of a more controlled efficacy study, which accounted for most of the findings and provided a clear picture of the effects of the app-program on sleep.^55^

## Data Availability

Raw data were generated at the Laboratory for Sleep and Consciousness Research at the University of Salzburg, Austria. Derived data supporting the findings of this study are available from the corresponding author upon reasonable request.

## Abbreviations

AI, artificial intelligence; BSI, Brief Symptom Inventory; CBT-I, cognitive behavioral therapy for insomnia; dCBT-I, digital cognitive behavioral therapy for insomnia; ECG, electrocardiography; EEG, electroencephalography; EMG, electromyography; EOG, electrooculography; HRV, heart-rate variability; ISI, Insomnia Severity Index; NOA, number of awakenings; PSG, polysomnography; PSQI, Pittsburgh Sleep Quality Index; SE, sleep efficacy; SOL, sleep onset latency; TST, total sleep time; T0, start of the study/baseline; T1, start of the 6-week training phase; T2, end of the 6-week training phase; T3, end of follow-up period after 4 weeks; WASO, wake after sleep onset; WHOQOL-Bref, WHO quality of life questionnaire (short version).
